# Association between service readiness and PMTCT cascade effectiveness: a 2018 cross-sectional analysis from Manica province, Mozambique

**DOI:** 10.1186/s12913-022-08840-3

**Published:** 2022-11-28

**Authors:** Aneth Dinis, Orvalho Augusto, Kristjana H. Ásbjörnsdóttir, Jonny Crocker, Sarah Gimbel, Celso Inguane, Isaías Ramiro, Joana Coutinho, Mery Agostinho, Emilia Cruz, Fernando Amaral, Esperança Tavede, Xavier Isidoro, Yaesh Sidat, Regina Nassiaca, Filipe Murgorgo, Fátima Cuembelo, Carmen E. Hazim, Kenneth Sherr

**Affiliations:** 1 National Directorate of Public Health, Ministry of Health of Mozambique, Maputo, Mozambique; 2 Department of Global Health, University of Washington, Seattle, USA; 3 Eduardo Mondlane University, Maputo, Mozambique; 4 Centre of Public Health Sciences, University of Iceland, Reykjavík, Iceland; 5 Department of Epidemiology, University of Washington, Seattle, USA; 6 Department of Child, Family & Population Health Nursing, University of Washington, Seattle, USA; 7 Comité para a Saúde de Moçambique, Chimoio, Mozambique; 8 Manica Provincial Health Directorate, Chimoio, Mozambique; 9 Department of Industrial and Systems Engineering, University of Washington, Seattle, USA

**Keywords:** PMTCT, Early infant HIV diagnosis, HIV PCR testing, Vertical HIV transmission, Service readiness, Mozambique

## Abstract

**Background:**

Despite high coverage of maternal and child  health services in Mozambique, prevention of mother-to-child transmission of HIV (PMTCT) cascade outcomes remain sub-optimal. Delivery effectiveness is modified by health system preparedness. Identifying modifiable factors that impact quality of care and service uptake can inform strategies to improve the effectiveness of PMTCT programs. We estimated associations between facility-level modifiable health system readiness measures and three PMTCT outcomes: Early infant diagnosis (polymerase chain reaction (PCR) before 8 weeks of life), PCR ever (before or after 8 weeks), and positive PCR test result.

**Methods:**

A 2018 cross-sectional, facility-level survey was conducted in a sample of 36 health facilities covering all 12 districts in Manica province, central Mozambique, as part of a baseline assessment for the SAIA-SCALE trial (NCT03425136). Data on HIV testing outcomes among 3,427 exposed infants were abstracted from at-risk child service registries. Nine health system readiness measures were included in the analysis. Logistic regressions were used to estimate associations between readiness measures and pediatric HIV testing outcomes. Odds ratios (OR) and 95% confidence intervals (95%CI) are reported.

**Results:**

Forty-eight percent of HIV-exposed infants had a PCR test within 8 weeks of life, 69% had a PCR test ever, and 6% tested positive. Staffing levels, glove stockouts, and distance to the reference laboratory were positively associated with early PCR (OR = 1.02 [95%CI: 1.01–1.02], OR = 1.73 [95%CI: 1.24–2.40] and OR = 1.01 [95%CI: 1.00–1.01], respectively) and ever PCR (OR = 1.02 [95%CI: 1.01–1.02], OR = 1.80 [95%CI: 1.26–2.58] and OR = 1.01 [95%CI: 1.00–1.01], respectively). Catchment area size and multiple NGOs supporting PMTCT services were associated with early PCR testing OR = 1.02 [95%CI: 1.01–1.03] and OR = 0.54 [95%CI: 0.30–0.97], respectively). Facility type, stockout of prophylactic antiretrovirals, the presence of quality improvement programs and mothers’ support groups in the health facility were not associated with PCR testing. No significant associations with positive HIV diagnosis were found.

**Conclusion:**

Salient modifiable factors associated with HIV testing for exposed infants include staffing levels, NGO support, stockout of essential commodities and accessibility of reference laboratories. Our study provides insights into modifiable factors that could be targeted to improve PMTCT performance, particularly at small and rural facilities.

**Supplementary Information:**

The online version contains supplementary material available at 10.1186/s12913-022-08840-3.

## Background

Transmission rates of HIV from an infected mother to her child range from 15 to 45% in the absence of any intervention; however, this can be reduced to less than 5% with antiretroviral therapy (ART) [1, 2]. Yearly, 1.3 million HIV infected women become pregnant worldwide, and an estimated 85% of them receive ART to prevent mother-to-child HIV transmission (PMTCT) [3]. Yet this observed high coverage in ART has not translated to elimination of MTCT, which is estimated to account for 90% of new HIV infections in children under 15 [4]. Although a reduction from 190,000 to 150,000 infections globally per year was documented between 2015 and 2020, this decrease is still far from the World Health Organization (WHO) target of zero new infant infections by 2030 [5, 6], and varies widely across highly affected countries [2].

Preventing vertical HIV transmission requires passing through a complex cascade that spans approximately two years and multiple biological periods for women-infant pairs; covers multiple services spanning antenatal care, institutional delivery, breastfeeding and pediatric support; and requires progression through each service stage to benefit from the linked steps across the PMTCT cascade [7–9]. Given that preventing vertical transmission requires successful completion of each PMTCT step – where progression through HIV testing, ART initiation and continuity with high adherence, safe delivery, adequate infant feeding, and infant antiretroviral prophylaxis is conditional on completion of previous cascade steps – services must be provided in a high quality and timely manner [10]. The complexity of the PMTCT cascade – including the fragmentation of services and actors and its conditional nature – impedes successful implementation of evidence-based PMTCT guidelines [8].

The WHO recommends that infants exposed to HIV during the intra- and peripartum periods be tested at 4–6 weeks of age or at the earliest opportunity beyond that time to optimize treatment strategies for infected infants [11, 12]. In 2018, however, only 59% of HIV-exposed infants globally received a virologic test by 2 months of age [13].

The prevalence of HIV in Mozambique is one of the highest in the world, with 13.2% of the adult population (15–49 years old) infected with HIV [14]. Estimates of progress toward Elimination of MTCT (EMTCT) as of 2020 from UNAIDS and the Mozambican Ministry of Health (MoH) indicate that coverage of early infant HIV diagnosis by 8 weeks was 83%, and the total MTCT rate across pregnancy, delivery and the breastfeeding period was 13% [15, 16]. In 2020, the number of new infections averted due to PMTCT in Mozambique is estimated at 20,000[13,000 – 34,000], with a lower transmission rate in infants tested before 8 weeks of life (5% vs. 16%) [16].

Health service delivery is greatly influenced by the robustness of health systems [17]. By identifying modifiable health systems factors that impact quality of care and service uptake, targeted strategies can be designed to optimize the delivery of health interventions [18]. To date the majority of research on determinants of PMTCT service performance (including early infant diagnosis) in sub-Saharan Africa relies on individual-level data to describe demographic predictors [19–21]. Limited evidence exists on the influence of supply-side determinants of PMTCT performance. We aimed to address this gap by investigating the influence of facility-level determinants on the performance of the PMTCT cascade, with a focus on early HIV testing in exposed infants. The identification of modifiable factors is important to recommend adaptations in service delivery and improve health outcomes.

## Methods

### Study design

We performed a cross-sectional study using baseline data from SAIA-SCALE (NCT03425136), a stepped-wedge trial ongoing in central Mozambique since 2018 to test the effectiveness of a novel method to disseminate a blended systems engineering implementation strategy to improve PMTCT services [22]. SAIA-SCALE is conducted in all 12 districts of Manica province in central Mozambique, and within each district, the three public health facilities with the largest volume of antenatal care patients were selected for inclusion [22]. Our analyses use baseline study data from these 36 health facilities (3 × 12 districts).

### Study setting

Manica province is in central Mozambique and has a population of 2 million [23]. The adult HIV prevalence is estimated at 13.5% [14]. Most health services are provided by the public sector. There are 121 public health facilities in Manica province, and the provider-to-population ratio is 6.4 physicians per 100,000 and 50.5 maternal & child health nurses per 100,000. Comparatively, the national ratio averages for both cadres are 8.7 and 53.6 per 100,000, respectively [23, 24]. There is high utilization of maternal & child services (MCHS) in Manica. Ninety-two percent of pregnant women receive at least one antenatal care (ANC) visit with a qualified professional, and 71% have an institutional delivery [14].

### Study population

Data were collected in 36 public sector health facilities as part of baseline data for the SAIA-SCALE project. Selection of facilities has been previously described [22]; briefly, all public sector primary care facilities providing PMTCT services were ranked by volume of first ANC visits in each district, and the three highest volume facilities selected for inclusion in the intervention trial. All 3427 women-infant pairs receiving PMTCT services in the 36 study health facilities between January and December 2017 were included in the study. The study population included all women who were diagnosed with HIV prior to seeking MCHS for this pregnancy, as well as those newly diagnosed through HIV testing during the pregnancy, delivery, or breastfeeding periods.

### Data sources

We used data from two sources. First, a baseline service readiness assessment modeled on the WHO Service Readiness and Availability Assessment (SARA) tool, was administered by study personnel in all 36 study facilities between May and August 2018. The SARA is a comprehensive, standardized tool from which we selected 9 measures to describe general conditions of health facilities, including detailed assessments of the availability of staffing, essential commodities, and medicines across relevant services in each facility (including antenatal care, maternity wards, postpartum care, at-risk childcare (CCR – *Consulta da Criança em Risco)*, pharmacy and laboratory) [25]. Second, we used data extracted from the CCR registries to track PMTCT indicators for HIV-exposed infants. CCR services provide care for children with elevated risk for illness, including HIV-exposed infants, who are targeted for HIV testing beginning at one month through 9 months age (virologic PCR test using dry blood spots for HIV-1), and administration of ART and prophylaxis for opportunistic infections depending on HIV status.

### Study Variables

#### Outcomes of interest

We examined three outcomes: 1) Whether the child had a PCR test by 8 weeks of life (EID or early infant diagnosis); 2) whether the child had a PCR test at any point before or after 8 weeks (ever PCR); and 3) whether the child had a positive HIV result by PCR before or after 8 weeks of life (positive PCR). All three outcomes were collected as binary measures at the mother-infant pair level from CCR registries, and aggregated into proportions of cases, across twelve months at the health facility level. Our final dataset included data from all 36 health facilities.

#### Facility characteristics

The following supply-side factors were included in the analysis: 1) personnel ratio (total number of clinical staff working in PMTCT services divided by the estimated population catchment area size of the facility); 2) health facility location (urban/rural); 3) stockouts for PMTCT commodities in the pharmacy in the last three months of a) pediatric antiretroviral nevirapine suspension (yes/no) and/or b) gloves (yes/no); 3) facility participation in a quality improvement program (yes/no), 4) presence of a mother-to-mother peer support group (yes/no); 5) number of NGOs supporting the facility (none/one/multiple); 6) catchment area population; and 7) driving distance from the health facility to the reference laboratory (kilometers). Three highly skewed variables were log-transformed for analysis: personnel ratio, catchment area size, and distance from health facility to reference laboratory.

### Statistical analysis

All analyses were conducted in R, version 4.1.2 [26], and at the health facility level (as no variables were available below this level). For each outcome the overall province-level proportion is pooled from facility-level proportions through a random-effects meta-analysis to account for and assess the heterogeneity between health facilities [27]. Univariate (additional file 2) and multivariable logistic regression models were used to assess the association between facility-level characteristics and the outcomes. Odds ratios (OR) and their 95% confidence intervals (95%CI) are reported. Because we included log-transformed covariates we provide guidance to interpret estimated coefficients in the appendix; briefly, we interpret these coefficients in terms of multiplicative change in odds per 5% increase in the predictor (additional file 1). For non-log transformed covariates we interpret the exponentiated coefficient as the OR.

## Results

### Study health facilities

Participating health facilities were predominantly rural (Table 1). Urban facilities had higher caregiver-to-patient ratios, lower prevalence of stockouts of prophylactic ARVs, lower number of NGO providing support, larger catchment areas and shorter distances to the reference laboratory compared with rural health facilities. Rural facilities had more quality improvement initiatives and mother-to-mother support groups (Table 1).

**Table 1:** Study health facilities characteristics by urbanicity

	**Rural HF** **(*N* = 24)** **N (%)**	**Urban HF** **(*N* = 12)** **N (%)**	**Total** **(*N* = 36)** **N (%)**
**Personnel ratio per 1000 inhabitants**			
Mean (SD)	0.20 (0.11)	0.24 (0.14)	0.21 (0.12)
**ARV stockout**^**a**^			
Yes	5 (21%)	2 (17%)	7 (19%)
No	19 (79%)	10 (83%)	29 (81%)
**Gloves stockout**^**a**^			
Yes	1 (4%)	9 (75%)	10 (28%)
No	23 (96%)	3 (25%)	26 (72%)
**Number of NGOs**			
None	0 (0%)	2 (17%)	2 (6%)
One	7 (29%)	4 (33%)	11 (31%)
Multiple	17 (71%)	6 (50%)	23 (64%)
**Mother-to-Mother Group**			
Yes	15 (62%)	12 (100%)	27 (75%)
No	9 (38%)	0 (0%)	9 (25%)
**QI program**			
Yes	23 (96%)	10 (83%)	33 (92%)
No	1 (4%)	2 (17%)	3 (8%)
**Catchment area (per 1000 people)**			
Mean (SD)	25000 (13000)	43000 (20000)	31000 (18000)
**Laboratory distance (Km)**			
Mean (SD)	63 (71)	24 (20)	50 (62)

*HF* Health facility, *ARV* Antiretroviral, *QI* Quality improvement, *NGOs* Non-Governmental Organizations

^a ^All stockouts are measured in the 3 months prior to the day of data collection

### HIV testing and diagnosis

**Fig. 1:**
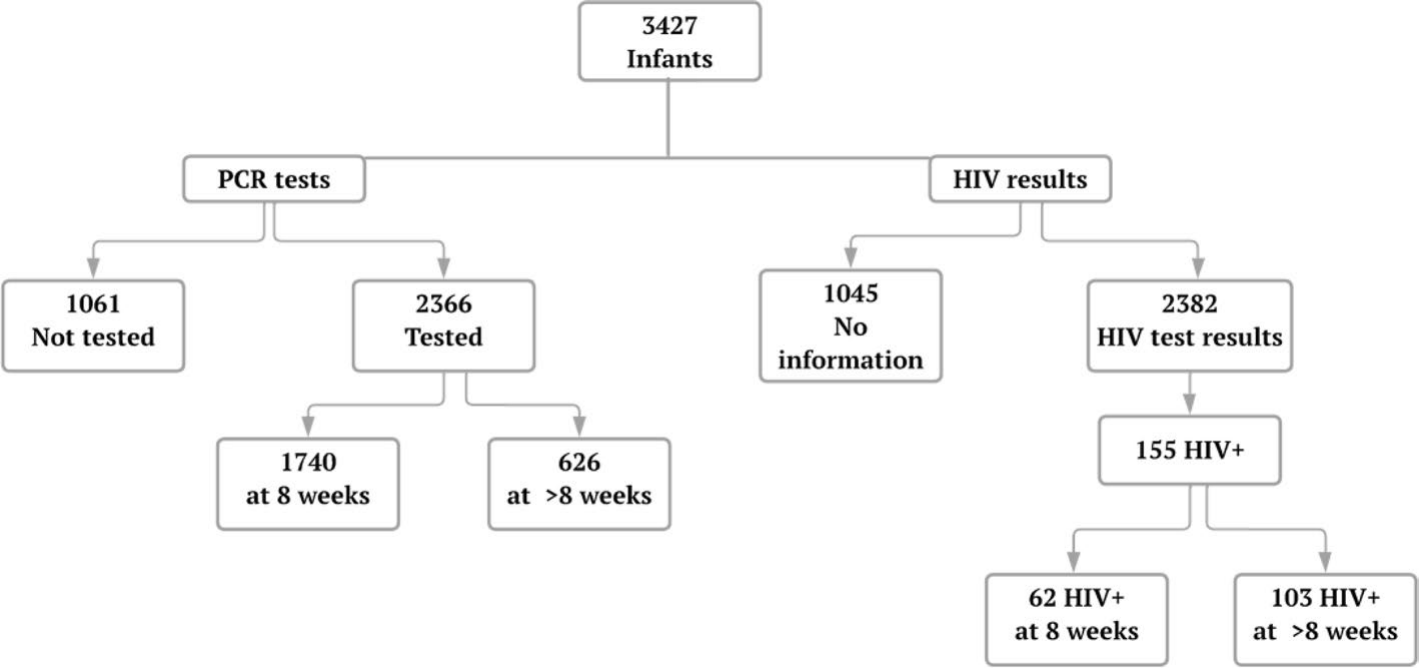
Number of infant HIV PCR tests and results in 36 facilities in Manica, 2017

Across the 36 study facilities, a total of 3,427 infants were identified as HIV exposed from pregnancy through the postpartum period, of whom 2,366 (69.0%) ever had a PCR test done and 2,382 (69.5%) had PCR results registered during the analysis period. Among PCR-tested infants, 1,740 (73.5%) were tested within 8 weeks of life. Over six percent of infants (n = 155, 6.5%) tested positive for HIV, the majority (n = 103, 66.5%) of whom were tested after 8 weeks of life (Fig. 1).

**Fig. 2:**
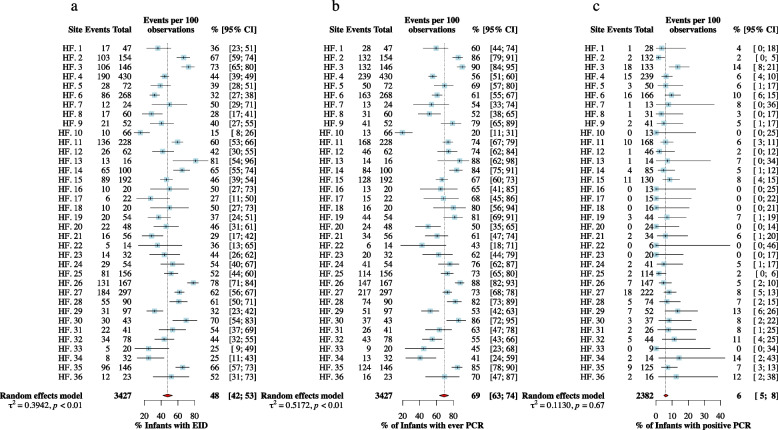
Facility-level and pooled proportions of study outcomes among HIV-exposed infants

The pooled proportion of infants with EID was 48% [95% CI: 42%, 53%], with noticeable heterogeneity between health facilities varying from 15% [95% CI: 8%, 26%] to 81% [95% CI: 54%, 96%] (Fig. 2a). The percentage of infants ever having a PCR test was 69% [95% CI: 63%, 74%], varying from 20% [95% CI: 11%, 31%] to 90% [95% CI: 84%, 95%] (Fig. 2b). The proportion of infants with a positive PCR HIV diagnosis was 6% [95% CI:5%, 8%], varying from range of 0% in eight health facilities to 14% in two facilities (Fig. 2c).

### Factors associated with early infant diagnosis (before 8 weeks of life)

In multivariable analysis caregiver-to-population ratio, stockout of gloves, support from multiple NGOs, catchment area size and distance from reference laboratory were significantly associated with EID (Table 2). For each 5% increase in the ratio of caregivers per 1000 people there was an associated 2% increase in odds for EID (OR = 1.02 [95%CI: 1.01–1.02]. Stockout of gloves in the pharmacy in the last 3 months was associated with 73% increased odds of EID (OR = 1.73 [95%CI: 1.24–2.40]). The involvement of multiple NGOs in the health facility was associated with 46% decreased odds of EID (OR = 0.54 [95%CI: 0.30–0.97]) compared with not having NGO support. A 5% increase in population catchment area was associated with 2% increase in odds for EID (OR = 1.02 [95%CI: 1.01–1.03] and each 5% increase in distance from a reference laboratory was associated with 1% increased odds of EID (OR = 1.01 [95%CI: 1.00–1.01]).

**Table 2:** Associations between health facility characteristics and early infant diagnosis

Variable	Adjusted Odds ratios (95%CI)	*P* value
Personnel/1,000 population^*^	1.02 [1.01–1.02] ^**†**^	< 0.0001
Facility Type (Urban)	1.14 [0.81–1.61]	0.4475
Stockout of ARVs (yes)	1.01 [0.79–1.27]	0.9650
Stockout of gloves (yes)	1.73 [1.24–2.40]	0.0011
QI program (yes)	1.52 [0.96–2.40]	0.0734
Mother-to-Mother program (yes)	1.21 [0.88–1.66]	0.2543
One NGO (ref: none)	0.61 [0.34–1.08]	0.0923
Multiple NGOs (ref: none)	0.54 [0.30–0.97]	0.0407
Catchment area size/1000^*^	1.02 [1.01–1.03] ^**†**^	< 0.0001
Distance from reference laboratory (km)^*^	1.01 [1.00–1.01] ^**†**^	0.0002

*QI* Quality improvement, *ARV* Antiretroviral (Nevirapine), *ref* Reference

^* ^Log transformed in the model

^† ^Transformed to odds ratio per 5% relative increase of the predictor to improve interpretability. Original coefficient for personnel ratio was 0.31 [95%CI: 0.18–0.43], for catchment area size was 0.39 [95%CI: 0.18–0.59] and for distance from the reference laboratory was 0.11 [95% CI: 0.07–0.15]

### Factors associated with ever having a PCR test (before or after 8 weeks of life)

The ratio of personnel, stockouts of gloves, and distance to the reference laboratory were significantly associated with ever having a PCR test (Table 3). For each 5% increase in the ratio of caregivers per 1000 patients there was an associated 2% increase in odds for ever having a PCR test (OR = 1.02 [95%CI: 1.01–1.02]). Stockout of gloves was associated with 80% increased odds for a PCR test (OR = 1.80 [95%CI: 1.26–2.58]). Each 5% increase in distance from a reference laboratory was associated with 1% increased odds of PCR (OR = 1.01 [95%CI: 1.00–1.01]).

**Table 3:** Associations between health facility characteristics and having a PCR test at any age

Variable	Adjusted Odds ratios (95%CI)	*P* value
Personnel/1,000 population^*^	1.02 [1.01–1.02]^**†**^	< 0.0001
Facility Type (Urban)	1.17 [0.81–1.70]	0.4006
Stockout of ARV (yes)	1.27 [0.98–1.64]	0.0773
Stockout of gloves (yes)	1.80 [1.26–2.58]	0.0012
QI program (yes)	1.32 [0.84–2.09]	0.2269
Mother-to-Mother program (yes)	1.12 [0.81–1.54]	0.4854
One NGO (ref: none)	0.91 [0.49–1.67]	0.7616
Multiple NGOs (ref: none)	0.87 [0.46–1.61]	0.6639
Catchment area size/1000^*^	1.01 [0.99–1.02]^**†**^	0.2144
Distance from reference laboratory (km)^*^	1.01 [1.00–1.01] ^**†**^	< 0.0001

*QI* Quality improvement, *ARV* Antiretroviral (Nevirapine), *ref* Reference

^* ^Log transformed in the model

^† ^Transformed odds ratio per 5% relative increase of the predictor to improve interpretability. Original coefficient for personnel ratio was 0.30 [95%CI: 0.16–0.43], for catchment area was 0.14 [95% CI: -0.08–0.35], and for distance from the reference lab was 0.13 [95%CI: 0.08–0.17]

### Factors associated with having a positive PCR HIV diagnosis

No covariates included in the analysis were found to be significantly associated with having a positive PCR HIV diagnosis (Table 4).

**Table 4:** Associations between health facility characteristics and positive infant PCR diagnosis

Variable	Adjusted Odds ratios (95%CI)	*P* value
Personnel/1,000 population^*^	1.01 [0.99–1.03] ^**†**^	0.2834
Facility Type (Urban)	1.46 [0.62–3.31]	0.3746
Stockout of ARV (yes)	1.02 [0.57–1.77]	0.9474
Stockout of gloves (yes)	0.78 [0.35–1.79]	0.5559
QI program (yes)	0.48 [0.18–1.27]	0.1281
Mother-to-Mother program (yes)	1.15 [0.48–3.09]	0.7693
One NGO (ref: none)	0.83 [0.25–3.27]	0.7729
Multiple NGOs (ref: none)	0.69 [0.21–2.75]	0.5692
Catchment area size/1000^*^	1.01 [0.98–1.03] ^**†**^	0.6027
Distance from reference laboratory (km)^*^	1.00 [0.99–1.00] ^**†**^	0.0663

*QI* Quality improvement, *ARV* Antiretroviral (Nevirapine), *ref* Reference

^* ^Log transformed in the model

^† ^Transformed to odds ratio per 5% relative increase of the predictor to improve interpretability. Original coefficient for personnel ratio was 0.21 [95%CI: -0.15–0.61], for catchment area was 0.14 [95%CI: -0.37–0.66] and for distance from the reference laboratory was -0.09 [95% CI: -0.20–0.01]

**Fig. 3:**
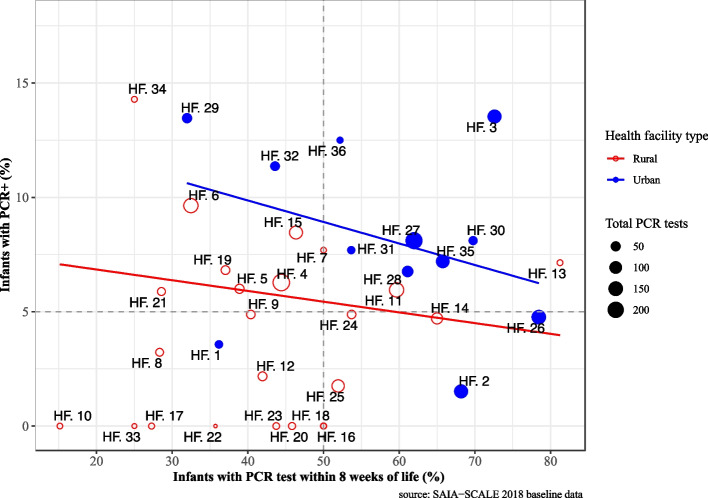
Relationship between facility-level EID performance and positive PCR diagnosis in 36 facilities

Figure 3 provides an exploratory visualization of the facility-level association between positive PCR tests and early infant testing, stratified by type of facility. The size of the plotted observations is proportional to the number of total PCR tests conducted and the unadjusted regression lines are weighted by the total number of PCR results by facility. Urban facilities tend to perform more early PCR tests compared to rural facilities and there is negative association between EID and positive PCR tests—for each 1% increase in the proportion of infants tested early, there is an 8.8% decrease in the proportion of positive PCR tests. For rural facilities, each 1% increase in the proportion of infants tested early there was a 4.8% decrease in the proportion of positive PCR tests. Although these directions are informative, they are not statistically significant.

## Discussion

National protocols for infant HIV diagnosis in Mozambique recommend that the first PCR test for HIV-exposed infants be conducted between 1–9 months of age, with subsequent rapid tests between 9–18 months for those with a negative PCR test through 9 months of age (with a second PCR test for confirmation of positive rapid tests) [28]. Programmatically, the MoH established 8 weeks of life as the target age for early infant diagnosis [28].

In exploratory analysis, without accounting for each facility’s contribution to the sample size, the overall proportion of tests conducted within 8 weeks, after 8 weeks, and proportion positive are 73.5, 26.5 and 6.5% respectively. However, to account for heterogeneity in sample size of the HFs and to be able to report confidence intervals, we estimated single and pooled proportions of the outcomes. Our exploration identified considerable heterogeneity in early PCR testing across the HFs included in this study, ranging from 15 to 81% of infants tested within 8 weeks of life and a pooled proportion of 48%. Comparatively, the MoH reported an average population coverage and Manica province coverage of PCR within 8 weeks of 66 and 70% respectively, in 2018 [29]. Our lower proportion might be attributable to the small sample of facilities in our study, with stratified selection of the largest facilities in each district, rather than the largest in the province overall.

The pooled proportion of PCR tests conducted increased to 69% when we consider both testing before and after 8 weeks of life, implying that at least 21% of all PCR tests were delayed beyond 8 weeks. The proportion of tests conducted after 8 weeks is comparable with the national average of 23% in 2018 [29]. The number of PCR test results in our sample exceeds the number of PCR tests performed (2,382 versus 2,366). A potential explanation for this difference is that some exposed infants are transferred to the CCR services with PCR tests already performed in other services (e.g., postpartum, or pediatric care) or other health facilities and then the results of these PCR tests are forwarded to the CCR services where the infant is being followed up.

We found an overall proportion of positive PCR diagnoses of 6%, though six facilities had greater than 10% positive PCR tests. It is important to note that some facilities with higher proportion of positive tests had a small number of total tests, suggesting that testing may have been prompted by symptomatic infection in some cases. Comparatively, the national average MTCT rate for 2018 was estimated at 9.9%; however, this rate is higher for infants tested after 8 weeks (16.9%) compared with those infants tested within 8 weeks (7.8%) [29].

Our results suggest that the caregiver-to-population ratio (a measure of workforce availability), glove stockouts and distance to the reference laboratory are associated with both EID and ever having been tested by PCR. Catchment area size and the involvement of multiple NGOs in the facility were associated with EID only (testing by 8 weeks of life). As hypothesized, increased staffing was associated with better performance in testing, but not with HIV transmission, as we suspect that individual patient-level factors continue to drive differential retention in care and ARV adherence, which in turn impacts viral suppression and the likelihood of vertical transmission [30, 31]. Counterintuitively, stockouts of gloves were associated with higher likelihood to perform PCR. A potential explanation for this is that having stockouts in the pharmacy does not necessarily mean that there are no gloves in specific services, as each service typically maintains their own small stock [as confirmed by our service readiness assessment (data not shown)]. In case of stockouts, health facility managers often prioritize gloves for priority and/or services that put health workers at increased risk, such as services with interactions with HIV-infected patients.

Larger facilities are frequently located close to reference laboratories, while small and rural facilities are required to send their blood samples for processing at distant laboratories [as confirmed by our service readiness assessment (data not shown)]. Our sample had more rural facilities than urban facilities (as is the case throughout Mozambique, where small, rural facilities greatly outnumber large, urban facilities), which may explain the positive association between PCR testing and distance to the reference laboratory. Larger catchment areas were found to be associated with EID [30], which is expected as larger catchment is associated with having larger facilities to meet the population needs, closer reference laboratories, and potentially higher likelihood of finding suspected cases compared with facilities serving smaller catchment areas [as confirmed by our service readiness assessment (data not shown)].

Contrary to our hypothesis, the presence of multiple NGOs supporting the facility, compared with not having any, was negatively associated with EID. A potential explanation is that having multiple NGOs working in the same location might lead to poor task coordination. This might particularly impact smaller facilities with few human resources, in which an overload of administrative tasks related to filling forms and reports for different projects may reduce available time to focus on clinical duties.

Surprisingly, facility urbanicity was not statistically associated with any of our outcomes, likely due to the small number of urban facilities compared with rural facilities. However, our visual exploration in Fig. 2 suggests that – overall – urban facilities tend to perform better than rural facilities.

Some results presented here align with published literature. In 2018 the WHO estimated that globally, only 56% of all HIV-exposed infants had access to EID by the second month of age [32], which is similar to our findings. A retrospective cohort study in a district hospital in Zambezia, Mozambique found that the median age for first PCR test for infants was 5 months (IQR, 2–7 months) and 16% among 105 infants tested positive [33]. However, this study was conducted in 2007–2008, prior to the introduction of Option B + and significant investments to expand PCR capacity. In Ethiopia a cohort study including 266 exposed infants found that 41% had had a PCR test within 6 weeks of life, and 13.2% of infants tested positive for HIV [34].

A systematic review that assessed accessibility of services for EID of HIV in sub-Saharan Africa, including Mozambique, found that stockout of supplies, weak infrastructures, inadequate human resources training and lack of sufficient reference laboratories with PCR capacity were ongoing challenges for delivering PMTCT [35]. Our results support the findings of this review.

There were some limitations to our study that merit caution in interpreting our results. First, this study was not designed to attribute causality. Second, the results are from a facility-level analysis and do not include individual-level determinants of PMTCT performance and vertical HIV transmission. Lastly, these results are from a relatively small sample of facilities in one province, and caution is merited in generalizing the results. Despite these limitations, there are notable strengths of our study: We used multiple data sources, the readiness data was collected with a standard tool, testing data was abstracted directly from patient registers, the study covers all districts in one province including urban and rural facilities, and facility-level findings are useful to inform local health managers in decision making.

## Conclusion

Facility-level modifiable factors can impact the delivery of PMTCT services. The availability of staffing, essential commodities, the size of population served, the involvement of NGOs and distance to the reference laboratory may influence PMTCT cascade outcomes. Program strategies can target these determinants of performance as a means to improve PMTCT performance.

## Supplementary Information

**Additional file 1.** Interpretation of log predictors: We explain how we transformed original odds ratios in log scale for three variables into more readily interpretable odds ratios.

**Additional file 2.** Unadjusted results for study outcomes: This file provides results from univariate analysis for all three outcomes. 

## Data Availability

The data that support the findings of this study are available upon reasonable request from the corresponding author and with permission of Manica provincial health directorate.

## Abbreviations

ART: Antiretroviral Therapy
ANC: Antenatal Care
CCR: Consulta da Criança em Risco (at-risk childcare in Portuguese language)
CI: Confidence intervals
EMTC: Elimination of Mother-to-Child Transmission
EID: Early Infant Diagnosis
HF: Health Facility
HIV: Human Immunodeficiency Virus
IQR: Interquartile Range
MTCT: Mother-to-Child Transmission
MoH: Ministry of Health
MCHS: Maternal and Child Health Services
NGOs: Non-Governmental Organizations
OR: Odds Ratio
PCR: Polymerase Chain Reaction
PMTCT: Prevention of Mother-to-Child Transmission
SARA: Service Readiness and Availability Assessment
WHO: World Health Organization
