# Supplementary material for: Association between service readiness and PMTCT cascade effectiveness: a 2018 cross-sectional analysis from Manica province, Mozambique
Source: BMC Health Serv Res. 2022 Nov 28;22:1422. doi: 10.1186/s12913-022-08840-3 (PMC9703771; doi:10.1186/s12913-022-08840-3)
Supplement: Supplementary file 1 — Additional file 1. Interpretation of log predictors: We explain how we transformed original odds ratios in log scale for three variables into more readily interpretable odds ratios. [file 12913_2022_8840_MOESM1_ESM.docx]

**Association between service readiness and PMTCT cascade effectiveness: A 2018 cross-sectional analysis from Manica province, Mozambique**

**Additional file 1**

**Interpretation of log-transformed predictor**

We included three covariates as log predictors in our models (distance from health facility to reference laboratory, catchment area population, and ratio of health workers to 1,000 inhabitants). Numerically, this parameterization improves the model; however, it poses challenges to interpret these coefficients. These are few notes to help interpret our results.

First, our regression models have the following general form:

$$log\left( odds \right)=\beta log\left( X_{1} \right)+\gamma X_{covariates}$$

Where:

$X_{1}$ is the predictor, whose coefficient we aim to interpret

$\beta$ is the coefficient to interpret

$X_{covariates}$ represents other covariates included in the models

$\gamma$ represents a vector of coefficients of the variable in $X_{covariates}$

We can rewrite the above equation as

$$\begin{matrix} odds=e^{\beta log\left( X_{1} \right)+\gamma X_{covariates}} \\ =e^{\beta log\left( X_{1} \right)}\cdot e^{\gamma X_{covariates}} \\ ={X_{1}}^{\beta}\cdot e^{\gamma X_{covariates}} \end{matrix}$$

So, if $X_{1}$ increases by a factor of $\kappa$ the odds would be multiplied by factor (i.e., the odds ratio):

$$\begin{matrix} OR=\frac{odds_{1}}{odds_{2}} \\ =\frac{\left( \kappa X_{1} \right)^{\beta}\cdot e^{\gamma X_{covariates}}}{{X_{1}}^{\beta}\cdot e^{\gamma X_{covariates}}} \\ =\kappa^{\beta} \end{matrix}$$

So, holding other covariates constant, an increase of $\kappa$ times (this is the same as $100\cdot\left( \kappa-1 \right)\%$) leads to $\kappa^{\beta}$ increase in the odds of the outcome.

In this report we choose $\kappa=1.05$ to interpret the odds ratios per 5% increase of the log-transformed predictor.
