# Supplementary material for: Association between service readiness and PMTCT cascade effectiveness: a 2018 cross-sectional analysis from Manica province, Mozambique
Source: BMC Health Serv Res. 2022 Nov 28;22:1422. doi: 10.1186/s12913-022-08840-3 (PMC9703771; doi:10.1186/s12913-022-08840-3)
Supplement: Supplementary file 2 — Additional file 2. Unadjusted results for study outcomes: This file provides results from univariate analysis for all three outcomes. [file 12913_2022_8840_MOESM2_ESM.docx]

**Association between service readiness and PMTCT cascade effectiveness: A 2018 cross-sectional analysis from Manica province, Mozambique**

**Additional file 2**

**Results from univariate analysis**

**Supplementary Table 1: Unadjusted associations between health facility characteristics and Early Infant Diagnosis (PCR testing within 8 weeks of life)**

| Variable | Unadjusted Odds ratios (95%CI) | P value |
| --- | --- | --- |
| Personnel/1,000 population^*^ | 1.01 [1.01-1.02] **^†^** | <0.0001 |
| Facility Type (Urban) | 2.07 [1.80-2.39] | <0.0000 |
| Stockout of ARV (yes) | 0.79 [0.66-0.96] | 0.0151 |
| Stockout of gloves (yes) | 2.27 [1.96-2.62] | 0.0000 |
| QI program (yes) | 1.73 [1.23-2.47] | 0.0020 |
| Mother-to-Mother program (yes) | 1.96 [1.52-2.55] | 0.0000 |
| One NGO (ref: none) | 0.85 [0.51-1.42] | 0.5446 |
| Multiple NGOs (ref: none) | 0.92 [0.56-1.52] | 0.7555 |
| Catchment area size^*^ | 1.02 [1.02-1.03] **^†^** | <0.0001 |
| Distance from reference laboratory (km)^*^ | 1.00 [1.00-1.00] **^†^** | 0.1562 |
| QI: Quality improvement; ARV: Antiretroviral (Nevirapine); ref: Reference.  ^*^Log transformed in the model.  ^†^Transformed to odds ratio per 5% relative increase of the predictor to improve interpretability. Original coefficient for personnel ratio was 0.27 [95% CI: 0.17-0.38], for catchment area was 0.45 [ 95% CI, 0.32; 0.59] and for distance from the reference laboratory was 0.03 [95% CI: -0.01-0.06] | | |

**Supplementary Table 2: Unadjusted associations between health facility characteristics and having a PCR test at any age**

| Variable | Unadjusted Odds ratios (95%CI) | P value |
| --- | --- | --- |
| Personnel/1,000 population^*^ | 1.01 [1.01-1.02] **^†^** | <0.0001 |
| Facility Type (Urban) | 1.93 [1.65-2.26] | <0.0000 |
| Stockout of ARV (yes) | 1.06 [0.86-1.30] | 0.6012 |
| Stockout of gloves (yes) | 2.18 [1.85-2.57] | <0.0000 |
| QI program (yes) | 1.56 [1.10-2.20] | 0.0114 |
| Mother-to-Mother program (yes) | 1.45 [1.12-1.86] | 0.0047 |
| One NGO (ref: none) | 1.18 [0.68-2.01] | 0.5385 |
| Multiple NGOs (ref: none) | 1.17 [0.68-1.95] | 0.5598 |
| Catchment area size^*^ | 1.01 [1.00-1.02] **^†^** | 0.0157 |
| Distance from reference laboratory (km)^*^ | 1.00 [1.00-1.01] **^†^** | 0.0032 |
| QI: Quality improvement; ARV: Antiretroviral (Nevirapine); ref: Reference.  ^*^Log transformed in the model.  ^†^ Transformed to odds ratio per 5% relative increase of the predictor to improve interpretability. Original coefficient for personnel ratio was 0.25 [95%CI: 0.15-0.36], for catchment area was 0.18 [95%CI: 0.03-0.32] and for distance from the reference laboratory was 0.06 [95%CI: 0.02-0.10] | | |

**Supplementary Table 3: Unadjusted associations between health facility characteristics and positive infant PCR diagnosis**

| Variable | Unadjusted Odds ratios (95%CI) | P value |
| --- | --- | --- |
| Personnel/1,000 population^*^ | 1.01 [0.99-1.02] **^†^** | 0.3601 |
| Facility Type (Urban) | 1.38 [1.00-1.91] | 0.0530 |
| Stockout of ARV (yes) | 0.96 [0.60-1.49] | 0.8742 |
| Stockout of gloves (yes) | 1.24 [0.89-1.71] | 0.2068 |
| QI program (yes) | 0.57 [0.29-1.23] | 0.1162 |
| Mother-to-Mother program (yes) | 1.65 [0.82-3.94] | 0.2066 |
| One NGO (ref: none) | 0.73 [0.27-2.52] | 0.5630 |
| Multiple NGOs (ref: none) | 0.64 [0.25-2.15] | 0.3969 |
| Catchment area size^*^ | 1.01 [0.99-1.03] **^†^** | 0.3115 |
| Distance from reference laboratory (km)^*^ | 1.00 [0.99-1.00] **^†^** | 0.0206 |
| QI: Quality improvement; ARV: Antiretroviral (Nevirapine); ref: Reference.  ^*^Log transformed in the model.  ^†^ Transformed to odds ratio per 5% relative increase of the predictor to improve interpretability. Original coefficient for personnel ratio was 0.13 [95% CI: -0.13-0.41],  for catchment area was 0.17 [95%CI: -0.16-0.51] and for distance from the reference laboratory was -0.103 [95% CI: -0.19 - -0.02] | | |
